# Genetic and environmental influences on eating behaviors in 2.5- and 9-year-old children: a longitudinal twin study

**DOI:** 10.1186/1479-5868-10-134

**Published:** 2013-12-07

**Authors:** Lise Dubois, Maikol Diasparra, Brigitte Bédard, Jaakko Kaprio, Bénédicte Fontaine-Bisson, Richard Tremblay, Michel Boivin, Daniel Pérusse

**Affiliations:** 1 Institute of Population Health, University of Ottawa, 1 Stewart St., Ottawa, ON, Canada; 2 Department of Epidemiology & Community Medicine, University of Ottawa, 451 Smyth Rd, Ottawa, ON, Canada; 3 Hjelt Institute, Department of Public Health, University of Helsinki, P.O. Box 41, Mannerheimintie 172, Helsinki, Finland; 4 National Institute for Health and Welfare, Department of Mental Health and Substance Abuse Services, P.O. Box 30, Mannerheimintie 166, Helsinki, Finland; 5 Institute for Molecular Medicine (FIMM), University of Helsinki, P.O. Box 20, Tukholmankatu 8, Helsinki, Finland; 6 Nutrition Sciences Program, University of Ottawa, 25 University Private, Ottawa, ON, Canada; 7 Research Unit on Children’s Psychosocial Maladjustment (GRIP), Université de Montréal, 3050 Édouard-Montpetit St., Montréal, QC, Canada; 8 School of Public Health, Physiotherapy & Population Science, University College Dublin, Dublin, Ireland; 9 École de psychologie, Université Laval, 2325 rue des Bibliothèques, Québec, QC, Canada; 10 Département d’anthropologie, Université de Montréal, C.P. 6128, succursale Centre-ville, Montréal, QC, Canada

**Keywords:** Eating behaviors, Heritability, Environmental influences, Children, Twins

## Abstract

**Background:**

Eating behaviors during childhood are related both to children’s diet quality and to their weight status. A better understanding of the determinants of eating behavior during childhood is essential for carrying out effective dietary interventions.

**Methods:**

We assessed the contribution of genetic and environmental factors to variations in selected eating behaviors in early and late childhood. Information on eating behaviors came from questionnaires administered to parents of children participating in the Quebec Newborn Twin Study when the twins were 2.5 and 9 years old (n = 692 children). Dichotomous variables were derived and analyzed using structural equation modeling, as part of a classic twin study design. We performed univariate and bivariate longitudinal analyses to quantify sources of variation and covariation across ages, for several eating behavior traits.

**Results:**

We found moderate to strong heritability for traits related to appetite such as eating too much, not eating enough and eating too fast. Univariate analysis estimates varied from 0.71 (95% CI: 0.49, 0.87) to 0.89 (0.75, 0.96) in younger children and from 0.44 (0.18, 0.66) to 0.56 (0.28, 0.78) in older children. Bivariate longitudinal analyses indicated modest to moderate genetic correlations across ages (*r*_A_ varying from 0.34 to 0.58). Common genetic influences explained 17% to 43% of the phenotypic correlation between 2.5 and 9 years for these appetite-related behaviors. In 9-year-old children, food acceptance traits, such as refusing to eat and being fussy about food, had high heritability estimates, 0.84 (0.63, 0.94) and 0.85 (0.59, 0.96) respectively, while in younger children, the shared environment (i.e., common to both twins) contributed most to phenotypic variance. Variances in meal-pattern-related behaviors were mostly explained by shared environmental influences.

**Conclusions:**

Genetic predispositions explain a large part of the variations in traits related to appetite during childhood, though our results suggest that as children get older, appetite-related behaviors become more sensitive to environmental influences outside the home. Still, for several traits environmental influences shared by twins appear to have the largest relative importance. This finding supports the notion that familial context has considerable potential to influence the development of healthy eating habits throughout childhood.

## Background

Childhood eating behaviors are attracting increasing interest in nutrition research because they embody various aspects of diet and are related to children’s diet quality and weight status [1-3]. Poor diet quality at a young age and deviations from optimal weight have been associated with an increased risk of developing chronic diseases later in life [4]. A better understanding of eating behavior determinants during childhood is of particular importance for those who plan and carry out dietary interventions and who develop recommendations for parents, schools and other caregivers.

A combination of genetic and environmental factors influences eating behaviors. Their relative influences vary by behavior and are likely to change across the life span, as is the case for phenotypes such as weight [5] and disordered eating symptoms during adolescence [6]. Studying monozygotic (MZ) and dizygotic (DZ) twin pairs is an effective way to investigate and quantify the contribution of genetic and environmental influences to variations in different eating traits. Moreover, twin studies can be used to control for genetic effects and thus further differentiate environmental influences related to life experiences that are shared or not shared by co-twins [7].

Eating behaviors include traits related to appetite, food acceptance and meal patterns. Existing twin studies have reported moderate to strong heritability for various appetitive traits in both infants and older children [8-10]. Neophobia, a trait linked to food acceptance, shows strong heritability in children aged 8 to 11 years [11]. Factors related to food intake regulation or taste predispositions could explain the relative importance of genetics in the expression of such eating traits [12-14]. Still, familial context and, as children get older, the environment outside the home may exert marked influences on some eating behaviors during childhood, including how eating is structured through meals and snacks [15]. However, few twin studies have investigated behaviors specifically related to eating context and meal patterns before adulthood. One exception is a study of breakfast frequency in 16-year-old adolescent twins, which suggested that family environment contributes substantially to variation in breakfast eating habits during adolescence, particularly among girls [16].

The number of twin studies on eating behaviors during childhood is limited, and most of them have been conducted in the United Kingdom [8-11]. Because eating behaviors can be complex, it is important both to replicate findings in other populations and to broaden the range of studied behaviors. Moreover, longitudinal analyses of twin data on children’s eating behaviors are rare and may enrich our understanding of how genetic and environmental influences evolve during childhood. Accordingly, one objective of our study is to quantify the genetic and environmental contributions to variations in selected eating behaviors related to appetite, food acceptance and meal patterns in early and late childhood. A second objective is to examine genetic and environmental correlations in these eating phenotypes throughout childhood and to quantify sources of covariation with age.

## Methods

### Participants

Information on eating behaviors in early and late childhood came from parents of children participating in the Quebec Newborn Twin Study (QNTS). This on-going cohort study of twins reared together and born between April 1, 1995, and December 31, 1998, in the Greater Montreal Area (Quebec, Canada) included 662 families (67% of eligible families) at the first assessment (when the children were 5 months old, adjusted for gestational age) [17]. Children with major diseases at birth were excluded from the cohort. Over the years, various aspects of the health and development of children participating in the study have been regularly assessed. As part of this longitudinal follow-up, information on several eating behaviors was collected when the children were 2.5 years old and later on, in a nutrition substudy, when they were 9 years old.

To determine the zygosity of the QNTS children, two independent raters assessed physical similarities when the twins were 5 months and 18 months old. Genetic marker analysis for a subsample of same-sex twins at both ages and chorionicity data from the twins’ medical files provided further confirmation, for an overall accuracy rate of 96%. More details on the method used to ascertain zygosity in the QNTS are available elsewhere [18].

### Measures

Information on children’s eating behaviors came from a questionnaire completed by a parent, generally the mother. An interviewer administered the questionnaire when the children were 2.5 years old; parents self-administered the questionnaire when the children were 9 years old. Selected characteristics of the sample are summarized in Table 1. Ethical approval was obtained from the Sainte-Justine Hospital Research Centre at each data collection point. The Ethics Committee at the University of Ottawa also approved the nutrition substudy conducted when the children were 9 years old. Parents gave their informed consent for participation at each data collection point.

**Table 1:** Sample characteristics

	**All twins**
	**(n = 692)**
Children’s sex	
Boys	48.0%
Girls	52.0%
Zygosity	
MZ	42.5%
DZ	57.5%
Children’s age (years)^1^	
T1	2.65 ± 0.08
T2	8.97 ± 0.54
Ethnicity (mother)	
White	89.9%
Non-white	6.4%
Ethnicity missing	3.8%
Parental education^2^ – T1	
No high school diploma	10.4%
High school diploma	7.2%
College diploma	35.3%
University diploma	41.9%
Education missing	5.2%
Annual family income ($Can) – T1	
< 20,000	9.2%
20,000-39,999	20.2%
40,000-59,999	22.5%
≥ 60,000	41.3%
Income missing	6.6%

^1^Mean ± SD.

^2^Represents the highest educational level for either parent.

Questions on eating behaviors were derived from questionnaires used in a similar cohort study of children (singletons) born in Quebec, the Quebec Longitudinal Study of Child Development (QLSCD 1998–2010). Most of these questions were based on questions in the Avon Longitudinal Study of Parents and Children (ALSPAC) [19], translated and modified slightly to reflect the context of the QLSCD. More details on the development of data collection instruments in the QLSCD are provided elsewhere [2,20].

Twelve questions pertaining to various appetitive traits, food acceptance traits and other behaviors related to meal patterns gathered information for deriving dichotomous variables for analysis. Table 2 presents the questions and response categories used to define these variables. Four questions about breakfast habits and the acceptance of vegetables, fruit and whole-grain bread were included only in the nutrition substudy at age 9. Dichotomous variables were defined according to theoretical and statistical considerations, that is to describe the extent to which parents perceived various eating behaviors as more or less problematic for their children while ensuring sufficient numbers for each category (yes versus no) to allow statistical analysis. Variables applicable to both younger and older children included “does not eat enough”, “eats too much”, “eats too fast”, “refuses to eat”, “fussy about food”, “eats at irregular hours”, “eats between meals”, and “eats a different meal”. Variables applicable only to 9-year-old children included “skips breakfast”, “fussy about vegetables”, “fussy about fruit”, and “fussy about whole-grain bread”.

**Table 2:** **Questions used to define eating behavior dichotomous variables**^**1**^

**Variable**	**Age**	**Question**	**Yes**	**No**
Does not eat enough	2.5; 9	In general, does your child eat enough?	Never; Rarely; Sometimes	Often
Eats too much	2.5; 9	In general, does your child over-eat?	Sometimes; Often	Never; Rarely
Eats too fast	2.5; 9	In general, does your child eat too fast?	Sometimes; Often	Never; Rarely
Refuses to eat	2.5; 9	In general, does your child refuse to eat?	Sometimes; Often	Never; Rarely
Fussy about food	2.5; 9	In general, is your child fussy about food?	Often	Never; Rarely; Sometimes
Eats at irregular hours	2.5; 9	In general, does your child eat at regular hours?	Never; Rarely; Sometimes	Often
Eats between meals	2.5; 9	In general, does your child eat between meals and so is not hungry at mealtime?	Sometimes; Often	Never; Rarely
Eats a different meal	2.5; 9	When your child is at home with you for the main meal of the day, how often does he/she eat a meal that is different from meals other family members eat?	Sometimes; Almost always; Always	Almost never
Skips breakfast	9	Does your child eat breakfast in the morning?	Regularly, but not every day; Only on occasion; Never	Yes, every morning
Fussy about vegetables	9	Refuses to eat vegetables when offered them OR Regularly eats vegetables (more than twice a week) with no problem	Very likely OR Not likely	Somewhat likely; not likely OR Very likely; Somewhat likely
Fussy about fruit	9	Refuses to eat fruit when offered it OR Regularly eats fruit (more than twice a week) with no problem	Very likely OR Not likely	Somewhat likely; not likely OR Very likely; Somewhat likely
Fussy about whole-grain bread	9	Refuses to eat whole-grain bread when offered it OR Regularly eats this kind of bread (more than twice a week) with no problem	Very likely OR Not likely	Somewhat likely; not likely OR Very likely; Somewhat likely

^1^Questions derived from questionnaires used in the Quebec Longitudinal Study of Child Development (QLSCD). Most of these questions were based on questions in the Avon Longitudinal Study of Parents and Children (ALSPAC) [19], translated and modified slightly to reflect the context of the QLSCD [20].

Except for variables related to the acceptance of vegetables, fruit and whole-grain bread, which combined responses to two similar but reversed items in multi-item questions, we analyzed questions first as ordinal variables based on four categories. Results of these analyses were close to those obtained using the dichotomous variables, suggesting that cutoffs for deriving our dichotomous variables were set appropriately and that bipartition adequately reflected the distribution of responses in our sample. For ease of interpretation, we presented results based on dichotomous variables.

### Statistical analysis

A total of 346 families provided information on children’s eating behaviors when their twins were aged 2.5 and 9 years. These families, with a total of 692 children, composed our sample for analysis. We controlled for birth order of twins through randomization at the time of data collection. Dichotomous variables were constructed as described in Table 2 and prevalence estimates (i.e., the percentage of children with a given trait) were computed and compared by sex, age and zygosity status.

To estimate how much genetic and environmental influences contributed to each eating behavior trait we used a twin study design that compared phenotypical resemblance between MZ and DZ twins. MZ twins typically share 100% of their segregating genes and DZ twins share 50% on average. Based on the assumption that MZ and DZ twins are equally exposed to similar environmental influences, a greater resemblance between MZ than DZ twins for a given phenotype indicates the presence of genetic influences on that trait [21-23]. Comparing intra-pair correlations between MZ and DZ twins thus constitutes a basis for analyzing phenotypic variance, which allowed us both to determine the etiology model that best explained variation in a trait and to quantify the relative contribution of genetic and environmental influences.

Classic twin study designs consider four components of phenotypic variance. These include two types of genetic influences: the cumulative effects of genes, known as additive genetic influences (A), and genetic effects resulting from interactions between alleles at the same locus (dominance) or different loci (epistasis), known as non-additive genetic influences (D). The other two components comprise environmental influences that are either shared by the twins or unique to one of the co-twins. More specifically, shared environmental influences (C) refer to factors or living experiences that have similar effects in both twins, making them more similar than expected from their genetic background. Unique environmental influences (E) refer to factors or living experiences that distinguish one twin from another in a family, thus making each twin unique in this respect. In analyses of phenotypic variance, the unique environment component also includes measurement error.

As part of these genetic analyses, we first estimated statistical measures of twin concordance and, in particular, the probandwise concordance rate and tetrachoric correlations. We defined the probandwise concordance rate as the probability that a twin would have a trait when the co-twin already has the trait. Tetrachoric correlations measure twin similarity as the likelihood of having a given trait. They assume that underlying each dichotomous measure is a normally distributed continuous variable.

For each dichotomous variable, the measures of twin concordance were separately calculated from 2×2 contingency tables for all twin pairs, zygosity types and zygosity-sex types. We used a chi-square test with one degree of freedom to determine differences in probandwise concordance rates between MZ and DZ twins. The McNemars test of symmetry allowed us to verify the equality of proportions derived from the marginal sums of the 2×2 contingency table. In addition, we compared tetrachoric correlations between MZ and DZ twins. These comparisons provided an initial indication of the presence of genetic influences and yielded insights as to which etiology model would be most appropriate for a given trait. For example, tetrachoric correlations (r) that are higher for MZ than for DZ twins indicate the presence of genetic influences; correlations that are higher for same-sex DZ twins than for opposite-sex twins indicate sex-specific influences. If rMZ = 2rDZ, an AE etiology model (i.e., without shared environmental influences) is likely to explain trait variation better. If rMZ < 2rDZ, given that rMZ > rDZ, an ACE model would appear to describe phenotypic variation better.

Alternatively, a model without genetic components (i.e., a CE model) would likely fit the data better if rMZ ~ rDZ. If rMZ > 2rDZ, then non-additive genetic influences (D) are likely to be present. Because C and D parameters cannot be estimated simultaneously in studies of twins reared together (like the present one), the potential for non-additive genetic influences to be present would suggest that an ADE model is preferable to an ACE model for investigating phenotypic variance. This was the case for the variables “does not eat enough”, “fussy about food” and “fussy about vegetables” in 9-year-old children, and “eats too much” and “eats too fast” at both ages. For all other variables under study, we looked to ACE models since correlations between MZ and DZ pairs tended to satisfy the inequalities 2rDZ > rMZ > rDZ.

We used equation modeling techniques [23,24] to ascertain which model best explained variations in specific eating behavior traits and to quantify the relative influences of the A, C (or D) and E variance components, when present. For each eating behavior trait, we tested basic assumptions about the equality of response (threshold) distributions within twin pairs and across zygosity. In all but one case, there was no significant change in model fit after equating thresholds within twin pairs and across zygosity. The only exception occurred for the “eats at irregular hours” trait in 2.5-year-old children. We therefore have not presented the results of equation modeling analysis for this trait in younger children. For each trait and age under study, we used liability threshold modeling techniques for twin data to fit different genetic models [23]. These models assume that the likelihood of having a trait is normally distributed.

We first built and compared a univariate saturated model and a sex-limited saturated model in order to determine if sex-specific influences were present [24,25]. Then we compared saturated and reduced models (ACE, AE, CE, E or ADE, AE, E). We also compared saturated and reduced models while controlling for the effects of several ordinal variables having to do with characteristics of children and their families (i.e., age, sex, ethnicity, parental education, annual family income). We determined the best-fitting models by taking into account both goodness-of-fit and parsimony in explaining the data. To this end, we considered the likelihood ratio test (−2LL) and Akaike’s Information Criterion (AIC: 2LL–2df) in selecting the best etiology model for a specific eating trait. The Bayesian Information Criterion (BIC: 2LL·ln[n], where n = sample size) was also used for comparison of basic and adjusted models. Standardized variance estimates of the A, D, C and E parameters in the best-fitting equation models were determined by the square of path coefficients (i.e., a^2^, d^2^, c^2^, and e^2^); 95% confidence intervals (CIs) were calculated.

For traits that were assessed both at 2.5 and 9 years, we calculated Pearson’s correlations to quantify the continuity in a specific behavior between 2.5 and 9 years. We used a bivariate ACE model applying Cholesky decomposition to examine further variance and covariance between these two ages for a given trait and to derive genetic and environmental correlations. These correlations indicate the extent to which the same genetic or environmental factors contribute to variation in a trait at both ages. As for variance, covariance can also be decomposed into genetic and environmental sources of influence. Bivariate models are then used to quantify the proportion of common influences on phenotypic correlations known as bivariate heritability and bivariate environmental influences (shared or unique) [23,24,26].

In the longitudinal analysis, we chose to examine ACE models since the univariate model-fitting with ADE models did not permit detection of a significant D component at both ages for a same eating trait (where applicable). This could be explained by a lack of power as fitting ADE models often require a larger sample size [27]. We first compared basic ACE models with models adjusted for age, sex or both age and sex. The lowest BIC determined the best bivariate ACE model, whether basic or adjusted. Then reduced models were compared to the full comparison model (i.e., the bivariate ACE model selected) and the best etiology model was selected based on lowest AIC and nonsignificant likelihood ratio chi-square test. From this final model we derived standardized variance and covariance estimates of the A, C and E parameters, genetic and environmental (shared and unique) correlations, and bivariate A, C and E, with their 95% confidence intervals (CIs). We performed basic statistical analyses using SAS version 9.3 (SAS Institute Inc., Cary, NC, USA) and genetic modeling analyses using OpenMx 1.3 library in R software [28]. In all cases, the level of significance was set at 0.05.

## Results

### Prevalence estimates

Table 3 presents prevalences for eating behavior traits under study. For children aged 2.5 years, estimates ranged from 8% for “eats at irregular hours” to 32% for “refuses to eat”. For those aged 9 years, estimates ranged from 8% for “skips breakfast” to 34% for “fussy about whole-grain bread”. Prevalences did not differ between MZ and DZ twins, and overall only a few differences emerged between boys and girls or between ages. The “refuses to eat” and “eats a different meal” traits were more prevalent among younger than older children. Conversely, the “eats too much” and “eats too fast” traits were more prevalent among older than younger children. The prevalence of “eats too fast” was also higher among boys than girls, while “eats at irregular hours” was higher among girls than boys.

**Table 3:** Prevalence estimates for selected eating behaviors

**Variable**	**Age**	***n *** **(twins)**	**%**
Does not eat enough	2.5	692	15.0
	9	690	14.3
Eats too much	2.5	692	11.4
	9	690	17.4^2^
Eats too fast	2.5	692	17.1
	9	692	26.7^2,3^
Refuses to eat	2.5	690	31.9^4^
	9	692	12.6
Fussy about food	2.5	690	9.4
	9	692	10.7
Eats at irregular hours	2.5	688	7.6
	9	692	8.5^5^
Eats between meals	2.5	692	27.9
	9	692	24.6
Eats a different meal	2.5	688	25.6^4^
	9	692	15.9
Skips breakfast	9	688	7.8
Fussy about vegetables	9	690^1^	18.7
Fussy about fruit	9	692	13.3
Fussy about whole-grain bread	9	534^1^	33.9

^1^Excluding families who indicated not offering the food item (first screening question).

^2^Prevalence higher in 9-year-old than in 2.5-year-old children (*P* < 0.05).

^3^Prevalence higher in boys than in girls (*P* < 0.05).

^4^Prevalence higher in 2.5-year-old than in 9-year-old children (*P* < 0.05).

^5^Prevalence higher in girls than in boys (*P* < 0.05).

### Measures of concordance

Comparison of MZ and DZ twins for probandwise concordance rates and for tetrachoric correlations indicated a similar pattern of results. We have, therefore, reported only tetrachoric correlations in Table 4. For several traits, we observed a tendency toward more concordance in MZ than DZ twins, suggesting the presence of genetic influences. More specifically, we noted significant differences in tetrachoric correlations for “does not eat enough” at both ages, for “eats too much” at age 2.5, and for “eats too fast”, “fussy about food”, “eats between meals” and “fussy about vegetables” at age 9. Conversely, for other traits such as “eats a different meal” and “fussy about whole-grain bread”, measures of concordance tended overall to be similar between MZ and DZ twins, suggesting that genetic factors did not contribute to phenotypic variance.

**Table 4:** Tetrachoric correlations between MZ and DZ twins for selected eating behaviors

**Variable**	**Age**	**n (twin pairs)**	**Tetrachoric correlations**	
			**MZ**	**DZ**
Does not eat enough	2.5	346	0.91 (0.81, 1.00)*	0.46 (0.19, 0.72)
	9	345	0.67 (0.45, 0.90)*	−0.09 (−0.43, 0.26)
Eats too much	2.5	346	0.88 (0.75, 1.00)*	0.43 (0.10, 0.75)
	9	345	0.52 (0.25, 0.80)	0.19 (−0.09, 0.47)
Eats too fast	2.5	346	0.72 (0.52, 0.92)	0.34 (0.08, 0.60)
	9	346	0.50 (0.27, 0.74)*	−0.02 (−0.27, 0.23)
Refuses to eat	2.5	346	0.85 (0.73, 0.96)	0.56 (0.39, 0.74)
	9	345	0.82 (0.64, 0.99)	0.47 (0.21, 0.73)
Fussy about food	2.5	346	0.84 (0.65, 1.00)	0.61 (0.37, 0.86)
	9	346	0.85 (0.68, 1.00)*	−0.02 (−0.38, 0.34)
Eats at irregular hours	2.5	346	0.98 (0.95, 1.00)	0.95 (0.86, 1.00)
	9	346	0.89 (0.74, 1.00)	0.74 (0.54, 0.95)
Eats between meals	2.5	344	0.96 (0.92, 1.00)	0.84 (0.73, 0.94)
	9	346	0.81 (0.68, 0.94)*	0.46 (0.24, 0.68)
Eats a different meal	2.5	344	1.00 (0.99, 1.00)	1.00 (0.99, 1.00)
	9	345	0.70 (0.48, 0.91)	0.71 (0.54, 0.88)
Skips breakfast	9	346	0.89 (0.73, 1.00)	0.77 (0.59, 0.95)
Fussy about vegetables	9	267	0.75 (0.57, 0.92)*	0.29 (0.02, 0.56)
Fussy about fruit	9	346	0.69 (0.46, 0.93)	0.50 (0.25, 0.76)
Fussy about whole-grain bread	9	345	0.80 (0.64, 0.95)	0.87 (0.78, 0.96)

*MZ is significantly different from DZ (*P* < 0.05). 95% CIs in parentheses.

### Best-fitting models

Table 5 presents standardized variance estimates for the best-fitting models from the univariate analysis. Details on the comparison of full ACE or ADE models with reduced models, including fit statistics, are available as additional tables (see Additional files 1, 2, 3 and 4). Models controlling for the effects of age, sex, ethnicity, parental education or annual family income did not provide a better fit than basic models, except for the “eats too fast” trait at age 9 where a sex-adjusted model provided the best fit (based on lowest BIC). A sex-limited model was also found to better explain variations in the “refuses to eat” trait at age 2.5. Therefore, in all but two cases, results reported from the univariate analysis refer to basic models.

**Table 5:** **Univariate analysis: standardized variance estimates of best-fitting models**^**1 **^**for selected eating behaviors**

**Variable**	**Age**	**Sex**	**Model**	**a**^**2**^	**d**^**2**^	**c**^**2**^	**e**^**2**^
Does not eat enough	2.5	Both	AE	0.89 (0.75, 0.96)	-	-	0.11 (0.04, 0.25)
	9	Both	AE	0.56 (0.28, 0.78)	-	-	0.44 (0.22, 0.72)
Eats too much	2.5	Both	AE	0.87 (0.70, 0.95)	-	-	0.13 (0.05, 0.30)
	9	Both	DE	-	0.55 (0.25, 0.77)	-	0.45 (0.23, 0.75)
Eats too fast	2.5	Both	AE	0.71 (0.49, 0.87)		-	0.29 (0.13, 0.51)
	9	Both	DE^2^	-	0.44 (0.18, 0.66)	-	0.56 (0.34, 0.82)
Refuses to eat	2.5	Girls	CE^3^	-	-	0.82 (0.65, 0.92)	0.18 (0.08, 0.35)
		Boys	ACE^3^	0.58 (0.16, 0.85)	-	0.22 (0.02, 0.53)	0.21 (0.07, 0.47)
	9	Both	AE	0.84 (0.63, 0.94)	-	-	0.16 (0.06, 0.37)
Fussy about food	2.5	Both	CE	-	-	0.70 (0.51, 0.84)	0.30 (0.16, 0.49)
	9	Both	DE	-	0.85 (0.59, 0.96)	-	0.15 (0.04, 0.41)
Eats at irregular hours	9	Both	CE	-	-	0.76 (0.58, 0.88)	0.24 (0.12, 0.42)
Eats between meals	2.5	Both	ACE	0.24 (0.02, 0.51)	-	0.71 (0.46, 0.89)	0.05 (0.01, 0.12)
	9	Both	AE	0.81 (0.66, 0.91)	-	-	0.19 (0.09, 0.34)
Eats a different meal	2.5	Both	CE	-	-	1.00 (0.99, 1.00)	0.00 (0.00, 0.01)
	9	Both	CE	-	-	0.70 (0.54, 0.81)	0.30 (0.19, 0.46)
Skips breakfast	9	Both	CE	-	-	0.82 (0.65, 0.92)	0.18 (0.08, 0.35)
Fussy about vegetables	9	Both	AE	0.73 (0.52, 0.87)	-	-	0.27 (0.13, 0.48)
Fussy about fruit	9	Both	CE	-	-	0.58 (0.38, 0.73)	0.42 (0.27, 0.62)
Fussy about whole-grain bread	9	Both	CE	-	-	0.84 (0.74, 0.91)	0.16 (0.09, 0.26)

^1^Based on lowest AIC and nonsignificant likelihood ratio chi-square test of model against saturated model (*P* > 0.05). 95% CIs in parentheses. Models adjusted for the effects of several ordinal variables (including age, sex, ethnicity, parental education and annual family income) did not provide a better fit than basic models (based on lowest BIC), except for *Eats too fast* at age 9 (see note 2).

^2^Model adjusted for children’s sex.

^3^Sex-limited model.

a^2^, proportion of variance explained by additive genetic influences; d^2^, proportion of variance explained by non-additive genetic influences; c^2^, proportion of variance explained by shared environmental influences; e^2^, proportion of variance explained by unique environmental influences, including measurement error.

At both ages, AE or DE models provided the best fit for “does not eat enough”, “eats too much” and “eats too fast” traits. Heritability estimates ranged from 0.71 (95% CI: 0.49, 0.87) to 0.89 (0.75, 0.96) in younger children and from 0.44 (0.18, 0.66) to 0.56 (0.28, 0.78) in older children, while the remaining influences were accounted for by the unique environment and measurement error. In 9-year-old children, the AE or DE models also better fit the data for “refuses to eat”, “fussy about food”, “fussy about vegetables”, and “eats between meals” traits. Heritability estimates varied from 0.73 (0.52, 0.87) to 0.85 (0.59, 0.96). An ACE model best explained phenotypic variance for the “eats between meals” trait in younger children. Even though the shared environment had a major relative influence (0.71; 0.46, 0.89), genetics contributed to most of the remaining influences (0.24; 0.02, 0.51). Similarly, an ACE model (sex-limited) provided the best fit for “refuses to eat” among boys aged 2.5 years. In this case, variance estimates indicated moderate heritability (0.58; 0.16, 0.85) and similar contributions from shared (0.22; 0.02, 0.53) and unique (0.21; 0.07, 0.47) environmental influences.

No evidence of genetic influences was detected in the variances for certain eating traits, at different ages, as shown by the best-fitting CE models. This was the case for “fussy about food” at age 2.5; for “eats at irregular hours”, “skips breakfast”, “fussy about fruit” and “fussy about whole-grain bread” at age 9; and for “eats a different meal” at both ages. For “refuses to eat” among girls aged 2.5 years, a CE model (sex-limited) fit the data best. In all cases, the shared environment contributed most to phenotypic variance, with relative influences ranging from 0.58 (0.38, 0.73) to 1.00 (0.99, 1.00). The unique environment, including measurement error, accounted for the remaining influences.

Seven eating behaviors of the univariate analysis were assessed both at age 2.5 and age 9. However, for “fussy about food” and “refuses to eat”, variance and covariance between ages could not be explained adequately by any of the bivariate longitudinal models. Therefore, results of the longitudinal analysis are presented for three appetite-related traits (i.e., “does not eat enough”, “eats too much” and “eats too fast”) and two meal-pattern-related behaviors (i.e., “eats between meals” and “eats a different meal”).

Table 6 presents standardized variance and covariance estimates of the best-fitting models in the bivariate longitudinal analysis. Details on the comparison between best bivariate ACE models and nested models, including fit statistics, are also available as additional tables (see Additional files 5 and 6). Overall, these results confirm findings of the univariate analysis for the contribution of genetic and environmental influences on the variance of these five eating traits in early and late childhood.

**Table 6:** **Bivariate longitudinal analysis: standardized variance and covariance estimates of best-fitting models**^**1,2 **^**for selected eating behaviors**

**Variable**	**Model**	**Estimate**	**a**^**2**^	**c**^**2**^	**e**^**2**^
Does not eat enough	AE (drop e_21_)	*Var*_2.5y_	0.91 (0.79, 0.97)	-	0.09 (0.03, 0.21)
		*Var*_9y_	0.62 (0.35, 0.82)	-	0.38 (0.18, 0.65)
		*Cov*_2.5-9y_	1.00 (1.00, 1.00)	-	-
Eats too much	AE (drop e_21_)	*Var*_2.5y_	0.87 (0.71, 0.95)	-	0.13 (0.05, 0.29)
		*Var*_9y_	0.52 (0.25, 0.74)	-	0.48 (0.26, 0.75)
		*Cov*_2.5-9y_	1.00 (1.00, 1.00)	-	-
Eats too fast	AE (drop e_21,_ sex_2.5_)	*Var*_2.5y_	0.71 (0.48, 0.87)	-	0.29 (0.13, 0.52)
		*Var*_9y_	0.38 (0.14, 0.60)	-	0.62 (0.40, 0.86)
		*Cov*_2.5-9y_	1.00 (1.00, 1.00)	-	-
Eats between meals	ACE (drop c_21_, e_21_)	*Var*_2.5y_	0.26 (0.06, 0.52)	0.69 (0.44, 0.86)	0.04 (0.01, 0.12)
		*Var*_9y_	0.72 (0.22, 0.91)	0.09 (0.00, 0.49)	0.20 (0.09, 0.37)
		*Cov*_2.5-9y_	1.00 (1.00, 1.00)	-	-
Eats a different meal	CE (drop e_21_)	*Var*_2.5y_	-	1.00 (0.99, 1.00)	0.00 (0.00, 0.01)
		*Var*_9y_	-	0.68 (0.56, 0.80)	0.32 (0.20, 0.46)
		*Cov*_2.5-9y_	-	1.00 (1.00, 1.00)	-

^1^Based on lowest AIC and nonsignificant likelihood ratio chi-square test of model against comparison model (*P* > 0.05) — See Additional files 5 and 6. 95% CIs in parentheses.

^2^All models refer to basic models (without adjustment for age or sex) except for *Eats too fast* trait for which a bivariate ACE model adjusted for children’s sex provided a better fit (lowest BIC).

c_21_, path coefficient of shared environmental influences present at age 2.5 on behavior trait at age 9; e_21_, path coefficient of unique environmental influences present at age 2.5 on behavior trait at age 9; sex_2.5y_, effect of sex at age 2.5; *Var*_2.5y_, proportion of variance explained by additive genetic influences (a^2^), shared environmental influences (c^2^) and unique environmental influences (e^2^) at age 2.5; *Var*_9y_, proportion of variance explained by a^2^, c^2^ and e^2^ at age 9; *Cov*_2.5-9y_, proportion of covariance explained by a^2^, c^2^ and e^2^ between 2.5 and 9 years.

Table 7 presents phenotypic, genetic and environmental correlations between the two ages for each of these five eating phenotypes. Phenotypic correlations (*r*) between 2.5 and 9 years, although low in magnitude (*r* varying from 0.13 to 0.22), remain significant, suggesting continuity to a certain extent in these five eating traits throughout childhood. We found modest to strong genetic correlations (*r*_A_) between these ages for the three appetite-related behaviors and for the “eats between meals” trait (*r*_A_ varying between 0.34 (0.07, 0.67) for “eats too fast” and 0.68 (0.33, 1.00) for “eats between meals”). These results suggest that the same set of genes partly influences these eating phenotypes both in early and late childhood. Our best etiological explanations or models for these traits did not allow any environmental correlations to be detected. Conversely, we found a moderate shared environmental correlation (*r*_C_) between ages for the “eats a different meal” trait (0.40; 0.24, 0.57), suggesting that the same factors from the shared environment partly influence this trait in early and late childhood.

**Table 7:** **Phenotypic, genetic and environmental correlations for selected eating behaviors between 2.5 and 9 years**^**1**^

**Variable**	***r***	***r***_**A**_	**Bivariate A**	***r***_**C**_	**Bivariate C**	***r***_**E**_	**Bivariate E**
Does not eat enough	0.22 (0.15, 0.29)	0.58 (0.37, 0.78)	0.43 (0.28, 0.57)	-	-	0.00	0.00
Eats too much	0.16 (0.09, 0.23)	0.43 (0.18, 0.69)	0.29 (0.12, 0.44)	-	-	0.00	0.00
Eats too fast	0.13 (0.05, 0.20)	0.34 (0.07, 0.67)	0.17 (0.03, 0.31)	-	-	0.00	0.00
Eats between meals	0.18 (0.11, 0.25)	0.68 (0.33, 1.00)	0.29 (0.16, 0.43)	0.00	0.00	0.00	0.00
Eats a different meal	0.17 (0.10, 0.25)	-	-	0.40 (0.24, 0.57)	0.33 (0.18, 0.46)	0.00	0.00

^1^From best-fitting models in the bivariate longitudinal analysis. 95% CIs in parentheses.

*r*, phenotypic correlation (Pearson’s correlation coefficient); *r*_A_, genetic correlation; *r*_C_, shared environmental correlation; *r*_E_, unique environmental correlation; Bivariate A, proportion of the phenotypic correlation between 2.5 and 9 years explained by common additive genetic influences; Bivariate C, proportion of the phenotypic correlation between 2.5 and 9 years explained by common influences from the shared environment; Bivariate E, proportion of the phenotypic correlation between 2.5 and 9 years explained by common influences from the unique environment.

As shown in Table 7, the bivariate heritability, that is the proportion of the phenotypic correlation between 2.5 and 9 years explained by common genetic influences at both ages, was found to be weak to moderate for the three appetite-related traits and for the “eats between meals” trait. Estimates varied from 0.17 (0.03, 0.31) to 0.43 (0.28, 0.57). For “eats a different meal”, the proportion of the phenotypic correlation between 2.5 and 9 years explained by common influences from the shared environment at both ages (bivariate C) was modest (0.33; 0.18, 0.46).

## Discussion

### Appetite-related traits

The present study found moderate to strong heritability for several eating traits in early and late childhood, as perceived by parents. These traits include behaviors that appear to reflect different dimensions of appetite, such as eating too much or not enough and eating too fast. Overall these findings agree with studies that have examined various appetite-related concepts in British twin children. For example, a study measuring eating rate in 254 twin children aged 10 to 12 years reported heritability estimates of 0.62 for this trait [8]. Heritability estimates of 0.63 and 0.75 were also reported for satiety responsiveness and food cue responsiveness, respectively, in children aged 8 to 11 years (n = 5435 twin pairs), based on parental responses to a psychometric questionnaire [9]. Using a similar instrument adapted for infants (n = 2402 twin pairs), the authors found appetitive traits to be moderately to strongly heritable at an early age, with heritability estimates ranging from 0.53 to 0.84 for the four constructs enjoyment of food, food responsiveness, satiety responsiveness and slowness in eating [10]. These converging results reinforce the notion that genetic predispositions explain a large part of the variations in traits related to appetite during childhood. It is also worth noting that in 9-year-old children, our results showed evidence for non-additive genetic effects in some eating traits.

A comparison of our variance estimates in 2.5- and 9-year-old children suggests that the relative influence of genetics decreases as children get older. The tendency toward decrease also appears in MZ correlations from young to older children for appetite-related traits. DZ correlations decrease even more for some variables. While genetic factors explain most of the variation in appetite-related traits in young children, the unique environment (including measurement error) tends to equal or even exceed genetics in its relative influence among older children. Such findings suggest that over the years, the children’s ability to regulate their appetite would become more sensitive to unique environmental influences. Factors outside the familial context might thus play an increasingly important role in quantity of food eaten and rate of eating among older children. Other studies have also suggested that the ability of children to self-regulate appetite differs by age, such that younger children would be less sensitive to various external cues (e.g., portion sizes) than older children [29].

At the phenotypic level, we noted modest continuity in appetite related behaviors between 2.5 and 9 years, which could partly reflect the long time interval between our assessments of eating behaviors. Other authors have reported modest to moderate continuity in appetite-related behaviors throughout childhood [30,31]. At the etiological level, our bivariate longitudinal analysis indicated modest to moderate genetic correlations for appetite-related traits, suggesting that the same genetic factors partly influence variations in a given trait both in early and late childhood. These common genetic influences would partly explain the continuity in appetite-related behaviors between 2.5 and 9 years.

### Food acceptance traits

In 9-year-old children, traits related to food acceptance, such as being fussy about food and refusing to eat, showed strong heritability. These results are consistent with the findings of a twin study examining neophobia in children aged 8 to 11 years [11]. In this latter study researchers administered a psychometric questionnaire to parents to assess this food acceptance trait in older children. They reported a heritability estimate of a magnitude similar to that in the present study, with no influence from the shared environment.

In younger children, our results revealed a different portrait of the relative influences that affect being fussy about food and, particularly among girls, refusing to eat. This finding appears to be in accordance with another twin study looking at “reaction to food” among various personality traits in young children (mean age 3.6 years) [32]. This earlier study did not detect genetic influences for this food acceptance trait, but it did detect strong shared environmental influences, thus suggesting that family environment contributes most to variations in food acceptance in early childhood. Overall, these findings are consistent with the central role that parents play in choosing what their children eat at a younger age. As children get older, they gain more autonomy in their food choices. They also have more opportunities to take meals outside the home. The relative influences of genetic predispositions on acceptance of various tastes and other sensory characteristics of foods would then become more prominent among older children. Differences between boys and girls in relative influence patterns for the “refuses to eat” trait in 2.5-year-old children are, however, more difficult to explain. Still, the wide confidence interval for heritability estimates for boys suggests that this result should be interpreted with caution.

Fussiness about vegetables, about fruit and about whole-grain bread, which was assessed only in 9-year-old children, showed variable patterns of relative influences. These patterns accord with the notion that determinants of food acceptance also vary according to the type of food offered to children. Being fussy about vegetables appeared to be highly heritable, just as the “refuses to eat” and “fussy about food” traits were heritable in older children. This finding is consistent with the existing literature, which shows associations for food choosiness, picky eating and food neophobia and a low acceptance of vegetables in childhood [33-35]. Genetic taste predispositions and particularly the natural propensity to reject bitter tastes, which are more present in vegetables in general, could explain the strong relative influence of genetic factors in this instance [36].

Conversely, environmental factors, particularly those shared by twins, appear to be stronger determinants of acceptance of fruit and whole-grain bread in 9-year-old children, although the unique environment also contributed quite substantially to variance for the “fussy about fruit” trait. It is worth noting that for younger children, a twin study conducted among preschoolers reported shared environment to have substantial relative influence on liking fruit and vegetables [37]. Overall, these findings tend to reinforce the relevance of strategies based on repeated exposure to specific foods and on their availability at home and elsewhere in children’s food environment as a means for promoting healthy food choices during childhood.

### Meal-pattern-related behaviors

Variances in meal-pattern-related behaviors were mostly explained by environmental influences, with the shared environment showing the largest contribution. The only exception was for the trait “eats between meals” in 9-year-old children, which showed strong heritability. In younger children we also detected some heritability, although the shared environment contributed most to variations in this behavior. It could be that in 9-year-old children, eating between meals is driven mostly by appetite, whereas in young children both eating frequency and meal and snack content are largely under parental control. This could explain differences in results between ages. For older children, eating between meals could relate more to appetitive traits, thereby giving genetics a greater relative influence. Still, bivariate longitudinal analysis indicated the presence of common genetic influences on this trait in early and late childhood, which partly explained the continuity in this behavior between 2.5 and 9 years.

Eating a meal different from meals eaten by other family members could be considered an indicator of food acceptance. Our results, however, suggest that it might better reflect how parents choose to respond to the child’s food acceptance or preferences and thus could relate more to familial influences over meal patterns. Longitudinal analysis indicated that common factors from the shared environment contribute to variations in this eating trait in early and late childhood and explain, to some extent, the continuity in this behavior. As for other traits related to meal patterns, our results reinforce the idea that familial context may exert a strong influence on eating habits [15,38].

### Strengths and limitations

A major strength of the present study on eating behaviors during childhood is that our analyses rely on information collected at two ages from children belonging to a relatively large population-based sample of twins reared together. Because we also looked at a number of behaviors and eating dimensions, we were able to assemble a spectrum of influences on eating habits that develop during childhood. Nevertheless, a larger sample size might have allowed detecting smaller genetic or shared environmental influences. For example, a lack of power might explain why we could not always detect a significant D component in some eating traits even though intraclass correlations suggested the presence of such genetic influences [27]. Our analysis of models controlling for a variety of child and family characteristics could also have benefited from a larger sample size. As our study is based on information provided by the same respondent for both twins, measurement error might be correlated between the twins, which could have inflated the shared environmental influences detected for some of the traits.

Some behaviors were assessed only when the children were aged 9 years, which limited age-based comparisons and longitudinal analyses. In addition, our assessment of eating behaviors is based on parental perceptions of children’s eating traits and relies on frequencies of responses to specific questions rather than on direct measurements or existing scales of various eating behavior constructs. Still, on the whole our results agree with those of other twin studies that have examined similar eating behavior concepts and thus support and confirm existing knowledge about how genetic and environmental influences contribute to various eating behavior traits in childhood. The next step will be to consider multivariate methods that are likely to improve our understanding of the complex etiologic pathways that characterize children’s eating behaviors. Gene-environment correlations and interactions may also be present and should be considered in future analyses.

## Conclusions

The present research contributes to the limited number of twin studies on eating behaviors during childhood. Such studies have great potential to enrich our understanding of the etiology of eating behaviors, especially during the early years when eating habits are being shaped. Our findings on eating behaviors in children aged 2.5 and 9 years reveal different patterns of relative influences from genetic and environmental factors according to type of eating behavior and age. In early childhood behaviors related to food intake volume thus seem to be most influenced by genetic predispositions that are potentially related to mechanisms of appetite regulation. In older children, although genetics still explains a substantial part of the variations in appetite-related behaviors, external influences outside the family environment also seem to play an important role. Nevertheless, genetic factors underlying variations in a given appetite-related trait were found to be partly the same in early and late childhood. These common genetic influences would explain, to some extent, the continuity of a specific eating behavior across ages.

As for food acceptance, the shared environment contributes substantially to variations in some eating traits, particularly in younger children who are under parental control for food choices and for specific types of food, such as fruit and whole-grain bread, as we found to be the case for 9-year-old children. However, in older children, who are more apt to choose the food they eat, being fussy about food in general and about vegetables in particular appears to be influenced largely by genetic taste predispositions. Finally, the shared environment influences meal-pattern-related behaviors for the most part. Overall these results reinforce the notion that the familial context and, later on, the environment outside the home can contribute substantially to eating habits during childhood and, in some cases, could modulate the expression of genetic predispositions [39].

## Abbreviations

A: Additive genetic influences; AIC: Akaike’s Information criterion; BIC: Bayesian Information criterion; DZ: Dizygotic twins; C: Shared environmental influences; D: Non-additive genetic influences; E: Unique environmental influences; MZ: Monozygotic twins; QLSCD: Quebec longitudinal study of child development; QNTS: Quebec newborn twin study; r: Phenotypic correlation; rA: Genetic correlation; rC: Shared environmental correlation; rE: Unique environmental correlation; SD: Standard deviation; 95% CI: 95% confidence interval.

## Competing interests

The authors declare that they have no competing interests.

## Authors’ contributions

LD was responsible for nutrition content of the QNTS, directed the analyses and interpretation of data, and had primary responsibility for final content of the manuscript. MD was responsible for all statistical analyses. BB and LD wrote the paper. JK provided guidance concerning data analysis and results interpretation in twin studies. BFB contributed to results interpretation. DP was responsible for recruitment of families in the QNTS. DP, RT and MB have been involved in the study design and follow-up of the cohort. All authors made critical revisions to the text and approved the manuscript submitted for publication.

## Supplementary Material

Additional file 1: Table S1 Results of the univariate model-fitting for selected appetite-related behaviors (including fit statistics). Click here for file

Additional file 2: Table S2 Results of the univariate model-fitting for selected food acceptance traits (including fit statistics). Click here for file

Additional file 3: Table S3 Results of the univariate model-fitting for selected meal-pattern-related behaviors (including fit statistics). Click here for file

Additional file 4: Table S4 Results of the sex-limited model-fitting for *Refuses to eat* at age 2.5 (including fit statistics). Click here for file

Additional file 5: Table S5 Comparison of selected bivariate ACE models for appetite-related behaviors between 2.5 and 9 years. Click here for file

Additional file 6: Table S6 Comparison of selected bivariate ACE models for meal-pattern-related behaviors between 2.5 and 9 years. Click here for file
