# Supplementary material for: Genetic and environmental influences on eating behaviors in 2.5- and 9-year-old children: a longitudinal twin study
Source: Int J Behav Nutr Phys Act. 2013 Dec 7;10:134. doi: 10.1186/1479-5868-10-134 (PMC4029536; doi:10.1186/1479-5868-10-134)
Supplement: Additional file 1: Table S1 — Results of the univariate model-fitting for selected appetite-related behaviors (including fit statistics). [file 1479-5868-10-134-S1.doc]

**Table S1 – Results of the univariate model-fitting^1,2^ for selected appetite-related behaviors**

| **Variable** | **Model** | **ep** | –**2LL** | **df** | Δχ^2^ | Δ df | *P* | **AIC** | **a^2^** | **d^2^** | **c^2^** | **e^2^** |
| --- | --- | --- | --- | --- | --- | --- | --- | --- | --- | --- | --- | --- |
| ***Does not eat enough*** |  |  |  |  |  |  |  |  |  |  |  |  |
| 2.5 years (*n*=346) | ACE | 4 | 525.60 | 689 | - | - | - | –852.40 | 0.87 | - | 0.03 | 0.11 |
|  | **AE** | **3** | **525.61** | **690** | **0.01** | **1** | **0.93** | –**854.39** | **0.89** | **-** | **-** | **0.11** |
|  | CE | 3 | 535.92 | 690 | 10.32 | 1 | 0.00 | –844.08 | - | - | 0.70 | 0.30 |
|  | E | 2 | 585.72 | 691 | 60.13 | 2 | 0.00 | –796.28 | - | - | - | 1.00 |
| 9 years (*n*=345) | ADE | 4 | 550.10 | 687 | - | - | - | –823.90 | 0.00 | 0.64 | - | 0.36 |
|  | **AE^1^** | **3** | **553.28** | **688** | **3.18** | **1** | **0.07** | –**822.72** | **0.56** | **-** | **-** | **0.44** |
|  | DE | 3 | 550.10 | 688 | 0.00 | 1 | 1.00 | –825.90 | - | 0.64 | - | 0.36 |
|  | E | 2 | 567.49 | 689 | 17.39 | 2 | 0.00 | –810.51 | - | - | - | 1.00 |
| ***Eats too much*** |  |  |  |  |  |  |  |  |  |  |  |  |
| 2.5 years (*n*=346) | ADE | 4 | 443.54 | 689 | - | - | - | –934.46 | 0.83 | 0.03 | - | 0.13 |
|  | **AE** | **3** | **443.55** | **690** | **0.00** | **1** | **0.96** | –**936.45** | **0.87** | **-** | **-** | **0.13** |
|  | DE | 3 | 444.88 | 690 | 1.34 | 1 | 0.25 | –935.12 | - | 0.87 | - | 0.13 |
|  | E | 2 | 491.50 | 691 | 47.96 | 2 | 0.00 | –890.50 | - | - | - | 1.00 |
| 9 years (*n*=345) | ADE | 4 | 625.57 | 687 | - | - | - | –748.43 | 0.20 | 0.33 | - | 0.46 |
|  | AE | 3 | 625.86 | 688 | 0.29 | 1 | 0.59 | –750.14 | 0.50 | - | - | 0.50 |
|  | **DE** | **3** | **625.69** | **688** | **0.12** | **1** | **0.73** | –**750.31** | **-** | **0.55** | **-** | **0.45** |
|  | E | 2 | 637.61 | 689 | 12.04 | 2 | 0.00 | –740.39 | - | - | - | 1.00 |
| ***Eats too fast*** |  |  |  |  |  |  |  |  |  |  |  |  |
| 2.5 years (*n*=346) | ADE | 4 | 603.47 | 689 | - | - | - | –774.53 | 0.59 | 0.13 | - | 0.28 |
|  | **AE** | **3** | **603.53** | **690** | **0.06** | **1** | **0.81** | –**776.47** | **0.71** | **-** | **-** | **0.29** |
|  | DE | 3 | 604.62 | 690 | 1.15 | 1 | 0.28 | –775.38 | - | 0.74 | - | 0.26 |
|  | E | 2 | 632.09 | 691 | 28.62 | 2 | 0.00 | –749.91 | - | - | - | 1.00 |
| 9 years (*n*=346) | ADE | 5 | 765.69 | 688 | - | - | - | –610.31 | 0.00 | 0.44 | - | 0.56 |
|  | AE | 4 | 767.07 | 689 | 1.37 | 1 | 0.24 | –610.93 | 0.39 | - | - | 0.61 |
|  | **DE** | **4** | **765.69** | **689** | **0.00** | **1** | **1.00** | –**612.31** | **-** | **0.44** | **-** | **0.56** |
|  | E | 3 | 776.28 | 690 | 10.59 | 2 | 0.01 | –603.72 | - | - | - | 1.00 |
| ^1^ Best model is in bold (based on lowest AIC and nonsignificant likelihood ratio chi-square test of model against saturated model; *P* > 0.05). The model with the second lowest AIC was selected as the best-fitting model for *Does not eat enough* at age 9 as validity of model fit was questionable for model with lowest AIC (lack of convergence).  ^2^ All models refer to basic models (without adjustment for control variables) except for *Eats too fast* at age 9 years (models adjusted for children’s sex).  ep, estimated parameters; –2LL, –2 log likelihood; df, degrees of freedom; Δχ^2^, change in chi-square test; Δdf, change in degrees of freedom; AIC, Akaike Information Criterion; a^2^, proportion of variance explained by additive genetic influences; d^2^, proportion of variance explained by non-additive genetic influences; c^2^, proportion of variance explained by shared environmental influences; e^2^, proportion of variance explained by unique environmental influences, including measurement error. | | | | | | | | | | | | |
