# Supplementary material for: Genetic and environmental influences on eating behaviors in 2.5- and 9-year-old children: a longitudinal twin study
Source: Int J Behav Nutr Phys Act. 2013 Dec 7;10:134. doi: 10.1186/1479-5868-10-134 (PMC4029536; doi:10.1186/1479-5868-10-134)
Supplement: Additional file 2: Table S2 — Results of the univariate model-fitting for selected food acceptance traits (including fit statistics). [file 1479-5868-10-134-S2.doc]

**Table S2 – Results of the univariate model-fitting^1,2^ for selected food acceptance traits**

| **Variable** | **Model** | | **ep** | –**2LL** | **df** | Δχ^2^ | Δ df | *P* | | **AIC** | **a^2^** | **d^2^** | | **c^2^** | | **e^2^** | |  |
| --- | --- | --- | --- | --- | --- | --- | --- | --- | --- | --- | --- | --- | --- | --- | --- | --- | --- | --- |
| ***Refuses to eat*** |  | |  |  |  |  |  |  | |  |  |  | |  | |  | |  |
| 9 years (*n*=346) | ACE | | 4 | 486.52 | 689 | - | - | - | | –891.48 | 0.72 | - | | 0.10 | | 0.17 | |  |
|  | **AE** | | **3** | **486.65** | **690** | **0.14** | **1** | **0.71** | | –**893.35** | **0.84** | **-** | | **-** | | **0.16** | |  |
|  | CE | | 3 | 491.42 | 690 | 4.90 | 1 | 0.03 | | –888.58 | - | - | | 0.62 | | 0.38 | |  |
|  | E | | 2 | 523.39 | 691 | 36.88 | 2 | 0.00 | | –858.61 | - | - | | - | | 1.00 | |  |
| ***Fussy about food*** |  | |  |  |  |  |  |  | |  |  |  | |  | |  | |  |
| 2.5 years (*n*=345) | ACE | | 4 | 393.43 | 687 | - | - | - | | –980.57 | 0.42 | - | | 0.40 | | 0.18 | |  |
|  | AE | | 3 | 395.22 | 688 | 1.79 | 1 | 0.18 | | –980.78 | 0.85 | - | | - | | 0.15 | |  |
|  | **CE** | | **3** | **395.13** | **688** | **1.70** | **1** | **0.19** | | –**980.87** | **-** | **-** | | **0.70** | | **0.30** | |  |
|  | E | | 2 | 430.77 | 689 | 37.34 | 2 | 0.00 | | –947.23 | - | - | | - | | 1.00 | |  |
| 9 years (*n*=346) | ADE | | 4 | 447.33 | 689 | - | - | - | | –930.67 | 0.00 | 0.85 | | - | | 0.15 | |  |
|  | AE | | 3 | 451.98 | 690 | 4.66 | 1 | 0.03 | | –928.02 | 0.77 | - | | - | | 0.23 | |  |
|  | **DE** | | **3** | **447.33** | **690** | **0.00** | **1** | **1.00** | | –**932.67** | **-** | **0.85** | | **-** | | **0.15** | |  |
|  | E | | 2 | 470.65 | 691 | 23.32 | 2 | 0.00 | | –911.35 | - | - | | - | | 1.00 | |  |
| ***Fussy about vegetables*** |  | |  |  |  |  |  |  | |  |  |  | |  | |  | |  |
| 9 years (*n*=345) | ADE | | 4 | 630.43 | 687 | - | - | - | | –743.57 | 0.73 | 0.00 | | - | | 0.27 | |  |
|  | **AE** | | **3** | **630.43** | **688** | **0.00** | **1** | **1.00** | | –**745.57** | **0.73** | **-** | | **-** | | **0.27** | |  |
|  | DE | | 3 | 630.61 | 688 | 0.18 | 1 | 0.67 | | –745.39 | - | 0.76 | | - | | 0.25 | |  |
|  | E | | 2 | 664.86 | 689 | 34.43 | 2 | 0.00 | | –713.14 | - | - | | - | | 1.00 | |  |
| ***Fussy about fruit*** | |  |  |  |  |  |  |  | |  |  |  | |  | |  | |  |
| 9 years (*n*=346) | | ACE | 4 | 514.26 | 689 | - | - | - | | –863.74 | 0.33 | - | | 0.33 | | 0.33 | |  |
|  | | AE | 3 | 515.52 | 690 | 1.26 | 1 | 0.26 | | –864.48 | 0.71 | - | | - | | 0.29 | |  |
|  | | **CE** | **3** | **515.12** | **690** | **0.86** | **1** | **0.35** | | –**864.88** | **-** | **-** | | **0.58** | | **0.42** | |  |
|  | | E | 2 | 542.46 | 691 | 28.20 | 2 | 0.00 | | –839.54 | - | - | | - | | 1.00 | |  |
| ***Fussy about whole-grain bread*** | | |  |  |  |  |  | |  |  |  | |  | |  | |  | |
| 9 years (*n*=267) | | ACE | 4 | 578.14 | 531 | - | - | - | | –483.86 | 0.00 | - | | 0.84 | | 0.16 | |  |
|  | | AE | 3 | 605.00 | 532 | 26.86 | 1 | 0.00 | | –459.00 | 0.87 | - | | - | | 0.13 | |  |
|  | | **CE** | **3** | **578.14** | **532** | **0.00** | **1** | **1.00** | | –**485.86** | **-** | **-** | | **0.84** | | **0.16** | |  |
|  | | E | 2 | 683.88 | 533 | 105.74 | 2 | 0.00 | | –382.12 | - | - | | - | | 1.00 | |  |
| ^1^Best model is in bold (based on lowest AIC and nonsignificant likelihood ratio chi-square test of model against saturated model; *P* > 0.05).  ^2^All models refer to basic models (without adjustment for control variables).  ep, estimated parameters; –2LL, –2 log likelihood; df, degrees of freedom; Δχ^2^, change in chi-square test; Δdf, change in degrees of freedom; AIC, Akaike Information Criterion; a^2^, proportion of variance explained by additive genetic influences; d^2^, proportion of variance explained by non-additive genetic influences; c^2^, proportion of variance explained by shared environmental influences; e^2^, proportion of variance explained by unique environmental influences, including measurement error. | | | | | | | | | | | | | | | | | |  |
