# Supplementary material for: Genetic and environmental influences on eating behaviors in 2.5- and 9-year-old children: a longitudinal twin study
Source: Int J Behav Nutr Phys Act. 2013 Dec 7;10:134. doi: 10.1186/1479-5868-10-134 (PMC4029536; doi:10.1186/1479-5868-10-134)
Supplement: Additional file 3: Table S3 — Results of the univariate model-fitting for selected meal-pattern-related behaviors (including fit statistics). [file 1479-5868-10-134-S3.doc]

**Table S3 – Results of the univariate model-fitting^1,2^ for selected meal-pattern-related behaviors**

| **Variable** | **Model** | **ep** | –**2LL** | **df** | Δχ^2^ | Δ df | *P* | **AIC** | **a^2^** | **d^2^** | **c^2^** | **e^2^** |
| --- | --- | --- | --- | --- | --- | --- | --- | --- | --- | --- | --- | --- |
| **Eats at irregular hours** |  |  |  |  |  |  |  |  |  |  |  |  |
| 9 years (*n*=346) | ACE | 4 | 360.22 | 689 | - | - | - | –1017.78 | 0.08 | - | 0.70 | 0.21 |
|  | AE | 3 | 366.07 | 690 | 5.85 | 1 | 0.02 | –1013.93 | 0.84 | - | - | 0.16 |
|  | **CE** | **3** | **360.30** | **690** | **0.08** | **1** | **0.78** | –**1019.70** | **-** | **-** | **0.76** | **0.24** |
|  | E | 2 | 403.34 | 691 | 43.12 | 2 | 0.00 | –978.66 | - | - | - | 1.00 |
| **Eats between meals** |  |  |  |  |  |  |  |  |  |  |  |  |
| 2.5 years (*n*=346) | **ACE** | **4** | **653.85** | **689** | **-** | **-** | **-** | –**724.15** | **0.24** | **-** | **0.71** | **0.05** |
|  | AE | 3 | 673.53 | 690 | 19.68 | 1 | 0.00 | –706.47 | 0.96 | - | - | 0.04 |
|  | CE | 3 | 658.48 | 690 | 4.63 | 1 | 0.03 | –721.52 | - | - | 0.89 | 0.11 |
|  | E | 2 | 819.21 | 691 | 165.36 | 2 | 0.00 | –562.79 | - | - | - | 1.00 |
| 9 years (*n*=346) | ACE | 4 | 712.59 | 689 | - | - | - | –665.41 | 0.68 | - | 0.12 | 0.20 |
|  | **AE** | **3** | **712.86** | **690** | **0.27** | **1** | **0.60** | –**667.14** | **0.81** | **-** | **-** | **0.19** |
|  | CE | 3 | 719.31 | 690 | 6.72 | 1 | 0.01 | –660.69 | - | - | 0.63 | 0.37 |
|  | E | 2 | 771.61 | 691 | 59.02 | 2 | 0.00 | –610.39 | - | - | - | 1.00 |
| **Eats a different meal** |  |  |  |  |  |  |  |  |  |  |  |  |
| 2.5 years (*n*=344) | ACE | 4 | 449.85 | 685 | - | - | - | –920.15 | 0.00 | - | 1.00 | 0.00 |
|  | AE | 3 | 550.12 | 686 | 100.27 | 1 | 0.00 | –821.88 | 1.00 | - | - | 0.00 |
|  | **CE** | **3** | **450.03** | **686** | **0.18** | **1** | **0.67** | –**921.97** | **-** | **-** | **1.00** | **0.00** |
|  | E | 2 | 782.44 | 687 | 332.59 | 2 | 0.00 | –591.56 | - | - | - | 1.00 |
| 9 years (*n*= 346) | ACE | 4 | 554.33 | 689 | - | - | - | –823.67 | 0.00 | - | 0.70 | 0.30 |
|  | AE | 3 | 563.69 | 690 | 9.36 | 1 | 0.00 | –816.31 | 0.78 | - | - | 0.22 |
|  | **CE** | **3** | **554.33** | **690** | **0.00** | **1** | **1.00** | –**825.67** | **-** | **-** | **0.70** | **0.30** |
|  | E | 2 | 606.11 | 691 | 51.78 | 2 | 0.00 | –775.89 | - | - | - | 1.00 |
| **Skips breakfast** |  |  |  |  |  |  |  |  |  |  |  |  |
| 9 years (*n*=344) | ACE | 4 | 326.94 | 685 | - | - | - | –1043.00 | 0.31 | - | 0.60 | 0.09 |
|  | AE | 3 | 332.66 | 686 | 5.72 | 1 | 0.02 | –1039.34 | 0.94 | - | - | 0.06 |
|  | **CE** | **3** | **328.45** | **686** | **1.51** | **1** | **0.22** | –**1043.55** | **-** | **-** | **0.82** | **0.18** |
|  | E | 2 | 378.49 | 687 | 51.55 | 2 | 0.00 | –995.51 | - | - | - | 1.00 |
| ^1^Best model is in bold (based on lowest AIC and nonsignificant likelihood ratio chi-square test of model against saturated model; *P* > 0.05).  ^2^All models refer to basic models (without adjustment for control variables).  ep, estimated parameters; –2LL, –2 log likelihood; df, degrees of freedom; Δχ^2^, change in chi-square test; Δdf, change in degrees of freedom; AIC, Akaike Information Criterion; a^2^, proportion of variance explained by additive genetic influences; d^2^, proportion of variance explained by non-additive genetic influences; c^2^, proportion of variance explained by shared environmental influences; e^2^, proportion of variance explained by unique environmental influences, including measurement error. | | | | | | | | | | | | |
