# Supplementary material for: Genetic and environmental influences on eating behaviors in 2.5- and 9-year-old children: a longitudinal twin study
Source: Int J Behav Nutr Phys Act. 2013 Dec 7;10:134. doi: 10.1186/1479-5868-10-134 (PMC4029536; doi:10.1186/1479-5868-10-134)
Supplement: Additional file 4: Table S4 — Results of the sex-limited model-fitting for Refuses to eat at age 2.5 (including fit statistics). [file 1479-5868-10-134-S4.doc]

**Table S4 – Results of the sex-limited model-fitting^1,2^ for *Refuses to eat*** at age 2.5

| **Model** |  | **ep** | –**2LL** | **df** | **Comparison model** | **Δχ^2^** | **Δ df** | *P* | **AIC** |
| --- | --- | --- | --- | --- | --- | --- | --- | --- | --- |
| 1 | ACE | 8 | 781.90 | 684 | - | - | - | - | –586.10 |
| 2 | AE | 6 | 788.56 | 686 | 1 | 6.66 | 2 | 0.04 | –583.44 |
| 3 | CE | 6 | 788.89 | 686 | 1 | 6.99 | 2 | 0.03 | –583.11 |
| 4 | E | 4 | 859.86 | 688 | 1 | 77.97 | 4 | 0.00 | –516.14 |
| **5** | **Partial ACE (drop a_f_)** | **7** | **781.89** | **685** | **1** | **0.00** | **1** | **1.00** | –**588.11** |
| 6 | Partial ACE (drop a_m_) | 7 | 783.77 | 685 | 1 | 1.87 | 1 | 0.17 | –586.23 |
| (*Continued: see columns below*) | | | | | | | | | |
|  | | | | | | | | | |

**Table A4 (*Continued*)**

|  |  | **Male** | | |  | **Female** | | |
| --- | --- | --- | --- | --- | --- | --- | --- | --- |
| **Model** |  | **a^2^** | **c^2^** | **e^2^** |  | **a^2^** | **c^2^** | **e^2^** |
| 1 | ACE | 0.58 | 0.22 | 0.21 |  | 0.00 | 0.82 | 0.18 |
| 2 | AE | 0.79 | - | 0.21 |  | 0.85 | - | 0.15 |
| 3 | CE | - | 0.55 | 0.45 |  | - | 0.77 | 0.23 |
| 4 | E | - | - | 1.00 |  | - | - | 1.00 |
| **5** | **Partial ACE (drop a_f_)** | **0.58** | **0.22** | **0.21** |  | **0.00** | **0.82** | **0.18** |
| 6 | Partial ACE (drop a_m_) | 0.00 | 0.72 | 0.28 |  | 0.54 | 0.31 | 0.15 |
| ^1^Best model is in bold (based on lowest AIC and nonsignificant likelihood ratio chi-square test of model against comparison model; *P* > 0.05).  ^2^All models constrain the shared environmental correlation (*r*_c_) to 1 and the additive genetic correlation (*r*_a_) to 0.5 for opposite-sex twins (DZOS).  ep, estimated parameters; –2LL, –2 log likelihood; df, degrees of freedom; Δχ^2^, change in chi-square test; Δdf, change in degrees of freedom; AIC, Akaike Information Criterion; a_f_ , path coefficient of additive genetic influences for girls; a_m_ , path coefficient of additive genetic influences for boys; a^2^, proportion of variance explained by additive genetic influences; c^2^, proportion of variance explained by shared environmental influences; e^2^, proportion of variance explained by unique environmental influences, including measurement error. | | | | | | | | |
