# Supplementary material for: Genetic and environmental influences on eating behaviors in 2.5- and 9-year-old children: a longitudinal twin study
Source: Int J Behav Nutr Phys Act. 2013 Dec 7;10:134. doi: 10.1186/1479-5868-10-134 (PMC4029536; doi:10.1186/1479-5868-10-134)
Supplement: Additional file 5: Table S5 — Comparison of selected bivariate ACE models for appetite-related behaviors between 2.5 and 9 years. [file 1479-5868-10-134-S5.doc]

**Table S5 - Comparison of selected bivariate ACE models^1,2^ for appetite-related behaviors between 2.5 and 9 years**

| Model |  | ep | –2LL | df | Comparison model | Δχ^2^ | Δ df | *P* | AIC |
| --- | --- | --- | --- | --- | --- | --- | --- | --- | --- |
|  | ***Does not eat enough*** |  |  |  |  |  |  |  |  |
| 1 | ACE | 11 | 1050.57 | 1375 | - | - | - | - | –1699.43 |
| 2 | AE | 8 | 1050.81 | 1378 | 1 | 0.24 | 3 | 0.97 | – 1705.19 |
| 3 | CE | 8 | 1068.38 | 1378 | 1 | 17.80 | 3 | 0.00 | – 1687.62 |
| **4** | **AE (drop e_21_)** | **7** | **1050.83** | **1379** | **2** | **0.01** | **1** | **0.90** | **–** **1707.17** |
|  |  |  |  |  |  |  |  |  |  |
|  | ***Eats too much*** |  |  |  |  |  |  |  |  |
| 1 | ACE | 11 | 1052.23 | 1375 | - | - | - | - | –1697.77 |
| 2 | AE | 8 | 1054.21 | 1378 | 1 | 1.98 | 3 | 0.58 | –1701.79 |
| 3 | CE | 8 | 1061.42 | 1378 | 1 | 9.19 | 3 | 0.03 | –1694.58 |
| **4** | **AE (drop e_21_)** | **7** | **1058.01** | **1379** | **2** | **3.80** | **1** | **0.05** | –**1699.99** |
| 5 | AE (drop a_21_ and e_21_) | 6 | 1068.99 | 1380 | 2 | 14.78 | 2 | 0.00 | –1691.01 |
|  |  |  |  |  |  |  |  |  |  |
|  | ***Eats too fast*** |  |  |  |  |  |  |  |  |
| 1 | ACE | 13 | 1360.05 | 1377 | - | - | - | - | –1393.95 |
| 2 | AE | 10 | 1360.05 | 1380 | 1 | 0.00 | 3 | 1.00 | –1399.95 |
| 3 | CE | 10 | 1368.05 | 1380 | 1 | 8.00 | 3 | 0.05 | –1391.95 |
| 4 | AE (drop e_21_) | 9 | 1362.78 | 1381 | 2 | 2.73 | 1 | 0.10 | –1399.22 |
| 5 | AE (drop a_21_ and e_21_) | 8 | 1368.93 | 1382 | 2 | 8.88 | 2 | 0.01 | –1395.07 |
| **6** | **AE (drop e_21_ and sex_2.5y_)** | **8** | **1364.65** | **1382** | **4** | **1.88** | **1** | **0.17** | –**1399.35** |
| ^1^Best model is in bold (based on lowest AIC and nonsignificant likelihood ratio chi-square test of model against comparison model; *P* > 0.05). ^2^ All models refer to basic models (without adjustment for age or sex) except for *Eats too fast* trait (models adjusted for children’s sex).  ep, estimated parameters; –2LL, –2 log likelihood; df, degrees of freedom; Δχ^2^, change in chi-square test; Δdf, change in degrees of freedom; AIC, Akaike Information Criterion; a_21_, path coefficient of additive genetic influences present at age 2.5 on behavior trait at age 9; e_21_, path coefficient of unique environmental influences present at age 2.5 on behavior trait at age 9; sex_2.5y_, effect of sex at age 2.5. | | | | | | | | | |
