# Supplementary material for: Genetic and environmental influences on eating behaviors in 2.5- and 9-year-old children: a longitudinal twin study
Source: Int J Behav Nutr Phys Act. 2013 Dec 7;10:134. doi: 10.1186/1479-5868-10-134 (PMC4029536; doi:10.1186/1479-5868-10-134)
Supplement: Additional file 6: Table S6 — Comparison of selected bivariate ACE models for meal-pattern-related behaviors between 2.5 and 9 years. [file 1479-5868-10-134-S6.doc]

**Table S6 - Comparison of selected bivariate ACE models^1,2^ for meal-pattern-related behaviors between 2.5 and 9 years**

| Model |  | ep | –2LL | df | Comparison model | Δχ^2^ | Δ df | *P* | AIC |  |
| --- | --- | --- | --- | --- | --- | --- | --- | --- | --- | --- |
|  | ***Eats between meals*** |  |  |  |  |  |  |  |  |  |
| 1 | ACE | 11 | 1348.38 | 1379 | - | - | - | - | –1409.62 |  |
| 2 | AE | 8 | 1368.22 | 1382 | 1 | 19.84 | 3 | 0.00 | –1395.78 |  |
| 3 | CE | 8 | 1359.80 | 1382 | 1 | 11.43 | 3 | 0.01 | –1404.20 |  |
| 4 | ACE (drop c_21_) | 10 | 1349.04 | 1380 | 1 | 0.66 | 1 | 0.42 | –1410.96 |  |
| 5 | ACE (drop e_21_) | 10 | 1349.05 | 1380 | 1 | 0.67 | 1 | 0.41 | –1410.95 |  |
| 6 | ACE (drop a_21_) | 10 | 1349.25 | 1380 | 1 | 0.87 | 1 | 0.35 | –1410.75 |  |
| 7 | ACE (drop a_21_, c_21_ and e_21_) | 8 | 1366.40 | 1382 | 1 | 18.02 | 3 | 0.00 | –1397.60 |  |
| 8 | ACE (drop a_21_ and c_21_)^3^ | 9 | 1358.93 | 1381 | 1 | 10.55 | 2 | 0.01 | –1403.07 |  |
| **9** | **ACE (drop c_21_ and e_21_)** | **9** | **1349.35** | **1381** | **1** | **0.97** | **2** | **0.62** | –**1412.65** |  |
| 10 | ACE (drop a_21_ and e_21_) | 9 | 1352.10 | 1381 | 1 | 3.72 | 2 | 0.16 | –1409.90 |  |
|  |  |  |  |  |  |  |  |  |  |  |
|  | ***Eats a different meal*** |  |  |  |  |  |  |  |  |  |
| 1 | ACE^3^ | 11 | 1009.54 | 1371 | - | - | - | - | –1732.46 |  |
| 2 | CE^3^ | 8 | 983.26 | 1374 | 1 | –26.28 | 3 | 1.00 | –1764.74 |  |
| **3** | **CE (drop e_21_)** | **7** | **985.15** | **1375** | **2** | **1.89** | **1** | **0.17** | –**1764.85** |  |
| 4 | CE (drop c_21_)^3^ | 7 | 997.92 | 1375 | 2 | 14.66 | 1 | 0.00 | –1752.08 |  |
| 5 | CE (drop c_21_ and e_21_)^3^ | 6 | 998.69 | 1376 | **2** | 15.43 | 2 | 0.00 | –1753.31 |  |
| ^1^Best model is in bold (based on lowest AIC and nonsignificant likelihood ratio chi-square test of model against comparison model; *P* > 0.05).  ^2^ All models refer to basic models (without adjustment for age or sex).  ^3^Validity of the model fit is questionable (lack of convergence).  ep, estimated parameters; –2LL, –2 log likelihood; df, degrees of freedom; Δχ^2^, change in chi-square test; Δdf, change in degrees of freedom; AIC, Akaike Information Criterion; a_21_, path coefficient of additive genetic influences present at age 2.5 on behavior trait at age 9; c_21_, path coefficient of shared environmental influences present at age 2.5 on behavior trait at age 9; e_21_, path coefficient of unique environmental influences present at age 2.5 on behavior trait at age 9. | | | | | | | | | |  |
